# Effectiveness of manual therapy and cervical spine stretching exercises on pain and disability in myofascial temporomandibular disorders accompanied by headaches: a single-center cohort study

**DOI:** 10.1186/s13102-023-00644-0

**Published:** 2023-03-23

**Authors:** In-su Lee, Suhn-yeop Kim

**Affiliations:** 1 Department of Physical Therapy, Graduate School, Daejeon University, Daejeon, Republic of Korea; 2 Department of Physical Therapy, College of Health and Medical Science, Daejeon University, 62 Daehak-ro, Dong-gu, Daejeon, 34520 Republic of Korea

**Keywords:** Cervical spine, Conservative therapy, Headache, Manual therapy, Neck pain, Temporomandibular disorders

## Abstract

**Background:**

Previous studies have demonstrated a relationship between headaches and temporomandibular disorders (TMDs). Moreover, recent studies have shown functional, anatomical, and neurological associations between the temporomandibular joint (TMJ) and upper cervical spine. This study aimed to investigate the effectiveness of manual therapy and cervical spine stretching exercises for pain and disability in patients with myofascial TMDs accompanied by headaches.

**Methods:**

Thirty-four patients recruited from Gyeryong Hospital with headaches and diagnosed with TMDs were randomly assigned to the experimental (n = 17) and control (n = 17) groups. Headache impact was assessed using the Korean Headache Impact Test-6. Masseter myofascial pain was measured using the visual analog scale, and TMJ pressure pain threshold levels were evaluated using an algometer. Neck pain intensity was assessed using the numerical rating scale. Once per week for 10 weeks, the experimental group received cervical spine-focused manual therapy and stretching exercises alongside conservative physical therapy, and the control group received conservative physical therapy alone. Patients were evaluated at baseline and 5 and 10 weeks post-intervention.

**Results:**

After the intervention, the experimental group exhibited significant reductions in the cervical kyphotic angle, Korean Headache Impact Assessment score, neck pain intensity, TMJ pain pressure threshold, Neck Disability Index score, and Jaw Functional Limitation Scale level compared with the control group (p < 0.01).

**Conclusion:**

Manual therapy and stretching exercises could help resolve TMDs accompanied by headaches through biomechanical changes in the cervical spine. These findings may guide protocols and clinical trials involving manual therapy that align morphological structures.

## Background

Temporomandibular disorder (TMD) is the second most common musculoskeletal disorder that causes pain and disability [1]. TMDs are associated with various abnormalities that promote structural and functional dysfunctions [2], which in turn result in symptoms and signs associated with the masticatory muscles and temporomandibular joint (TMJ) [3]. TMDs primarily prevent chewing, swallowing, and speaking. The most common symptom is pain in the upper portion of the face during mastication [4]. Several clinical and epidemiological studies have reported an association between headaches and TMDs through the efforts of TMJ specialists who develop the best evidence-based paradigms by integrating clinical reasoning with up-to-date neurophysiological evidence [5, 6].

Recently, functional, anatomical, and neurophysiological relationships have been reported between the TMJ and upper cervical spine. Associations between the cervical spine, TMDs, and head and neck conditions, such as neck pain (NP) and headaches, are widely recognized [7]. Gonçalves et al. [8] reported that individuals with myofascial TMDs are more likely to experience chronic daily headaches than those without myofascial TMDs. Similarly, trend analysis for pain-stricken TMD groups associated with an increased frequency of temporal headaches revealed considerable exacerbation of all signs and symptoms of TMDs [9]. Ferrillo et al. [10] reported a higher prevalence of headaches in patients with myogenous TMD. The prevalence of these TMDs is higher in chronic primary pain conditions related to central nervous system dysfunction, including fibromyalgia and primary headaches, probably through the phenomenon of central sensitization (mainly allodynia and hyperalgesia) [11].

The association between NP and TMD has also been studied extensively [10]. There is evidence regarding the relationship between cervical spine impairment and TMDs, as shown by a significant correlation between jaw and neck disabilities (r = 0.82) and the finding of significantly greater pain during neck movement in patients with TMDs than in asymptomatic persons [12].

Moreover, patients with TMDs exhibit reduced neck flexor and extensor muscle endurance [13]. The authors previously analyzed TMJ pain, TMD disability levels, and the severity of the cervical kyphotic angle and reported that greater TMD disability was associated with an increased cervical kyphotic angle [14].

Catanzariti et al. [15] reported that patients with NP may respond to interventions applied to the TMJ and that techniques applied to the cervical spine may also affect the TMJ. Manual therapy can be classified into the following three main types: manipulation, mobilization, and soft tissue (muscle energy) approaches, with a wide variety of associated techniques [16]. Research suggests that manual therapy for TMD could be beneficial in targeting the oral facial region and cervical spine [16]. The purpose of stretching is to restore the efficiency of lubrication and stimulate the synthesis of glycol-aminoglycan between collagen fibers that allow movement in the periarticular structures. Stretching exercises are beneficial for both arthrogenous and myogenous TMDs [17]. Therefore, manual therapy and stretching for the cervical spine could increase the efficacy of interventions in patients with TMDs or those who cannot undergo manual therapy to the mandible. Notably, various authors considered it appropriate to apply the same type of manual therapy to the cervical spine area, particularly the upper cervical spine, with a TMD-focused approach [18].

Regarding the association between the neck, headache, and TMJ, it can be hypothesized that interventions targeting the cervical spine may also affect patients with TMD-related headaches. Therefore, we explored the effects of cervical spine-focused manual therapy on headaches and cervical pain intensities, TMJ pressure pain threshold (PPT), cervical kyphotic angle, Neck Disability Index (NDI), and Jaw Functional Limitation Scale score (JFLS) without administering medications to patients with myofascial TMD accompanied by headaches over a specific period.

## Methods

### Aim

This study aimed to investigate the effectiveness of manual therapy and cervical spine stretching exercises for pain and disability in patients with myofascial TMD accompanied by headaches.

## Study participants

Patients who experienced headaches and were diagnosed with one or more symptoms of TMD (such as TMJ pain, mouth-opening restrictions, and TMJ clicks) at the Gyeryong Hospital (Gyeryong-si, Chungcheongnam-do, South Korea) from July to September 2022 were included in this study. The required sample size was calculated based on Cohen’s sampling formula using the G-power program (G*Power ver. 3.1.9.4; University of Kiel, Kiel, Germany). The effect size, significance level, and power were set to 0.25, 0.8, and 0.8, respectively. The inclusion and exclusion criteria were based on the study of La Touche et al. [18].

The inclusion criteria were as follows: (1) headaches experienced after the diagnosis of TMD, accompanied by one or more symptoms such as mouth-opening restriction, TMJ pain, and TMJ clicks; (2) a Korean Headache Impact Test-6 (KHIT-6) score of ≥ 50 points indicating the need for medical care [19]; (3) ability to participate in the experiment (from 24 h before the assessment and during the intervention period) without using analgesics or muscle relaxants; (4) met the primary Diagnostic Criteria for TMD (DC/TMD) Axis I, Group I: muscle disorders (including myofascial pain with and without mouth opening limitation) [20]; (5) a pain intensity of ≥ 30 mm in the masseter muscle fascia on the 100-mm visual analog scale (VAS), according to the diagnostic criteria described by Bergman [21]; (6) a history of pain of at least three months before the study; and (7) understanding of the purpose of the study and provision of written informed consent.

The exclusion criteria were as follows: (1) the DC/TMD Axis I, Group II: including disc displacement with or without reduction and mouth opening limitation; or Group III: arthralgia, arthritis, and arthrosis [20]; (2) a history of traumatic injury to the mandible or neck; (3) diagnosis of a systemic disease (rheumatoid arthritis, systemic iris lupus, or psoriasis arthritis); (4) fibromyalgia syndrome; (5) nervous system disorders, such as trigeminal neuralgia; (6) history of any form of treatment (such as physical therapy, splint therapy, acupuncture, or Botox treatment) within three months preceding the study; (7) inability to stand upright for radiographic evaluation; and (8) congenital deformities of the head and neck areas.

## Procedures

The study’s purpose and procedures were explained to the participants. Subsequently, only those who provided written consent to participate were included. The patients were evaluated using a pre-prepared questionnaire to determine eligibility. To meet the inclusion criteria, after ruling out dental pain, patients who reported referred pain in response to masticatory muscle palpation were diagnosed as having masticatory myofascial pain. Patients who met the criteria for arthralgia and/or intraarticular TMD were excluded.

**Fig. 1:**
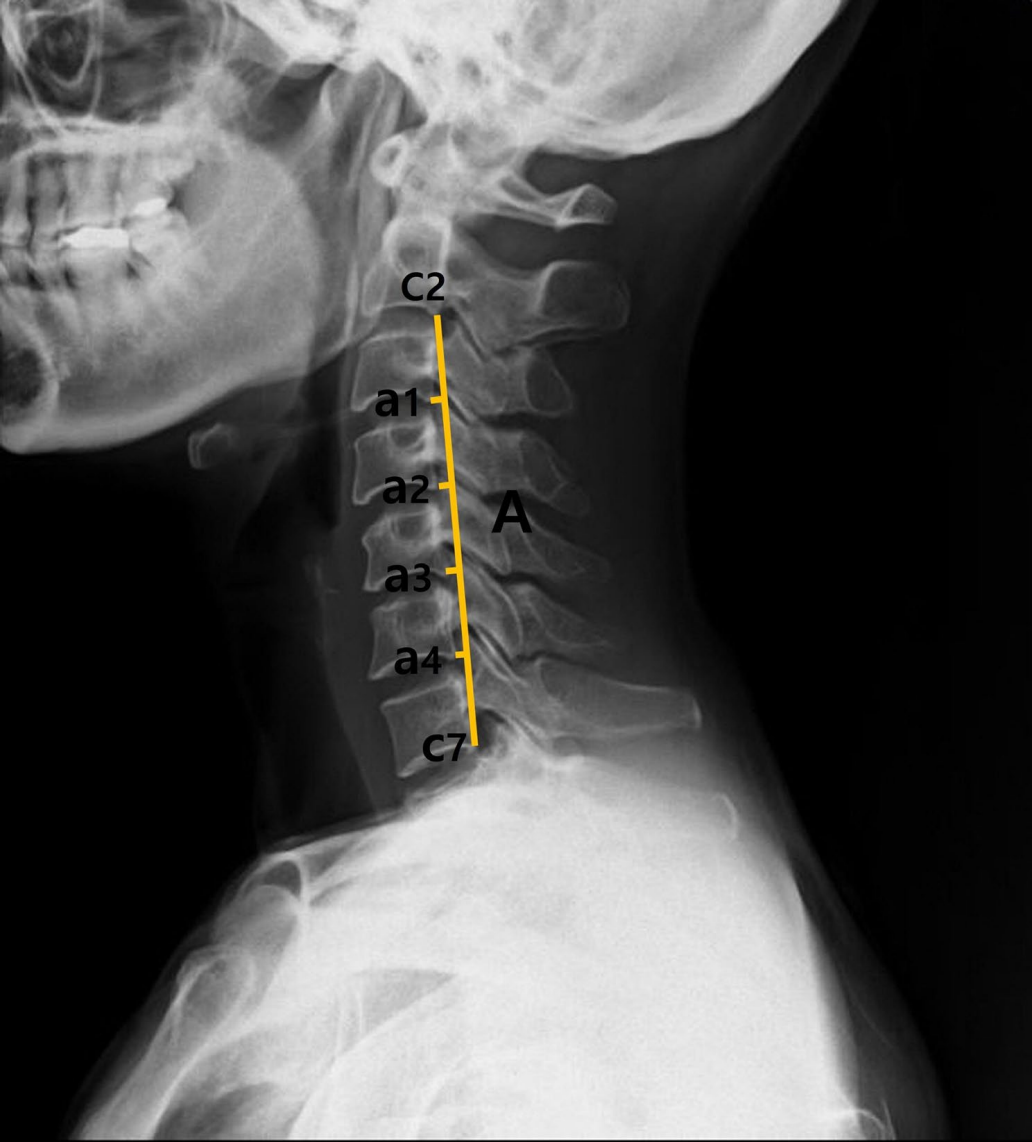
Study flow diagram

First, the Headache Impact Assessment Questionnaire was used to select patients with a KHIT-6 score of ≥ 50 points, indicating the need for medical care [19]. The masseter muscle fascia was measured by the VAS after the cervical kyphotic angle was measured radiographically. Further, the neck disability level, jaw functional limitation, and TMJ PPT levels were evaluated. Active participation was encouraged through wired and wireless telephone consultations. The study design is illustrated in Fig. 1. The study was conducted after obtaining approval from the Agency Bioethics Committee of Daejeon University during the design phase (Approval No. 1040647-202006-HR-003).

## Evaluation methods and measurement tools

### Headache intensity: KHIT-6

The Korean version of the Headache Impact Assessment Questionnaire standardized by Chu et al. [19] was used to assess headache intensity in patients. It comprises six questions that evaluate social functioning, role functioning, cognitive functioning, psychological distress, and vitality. Each question contains six items, each with five response options (never, 6; rarely, 8; sometimes, 10; very often, 11; and always, 13 points). Total scores of 36 and 78 indicate mild and excruciating headaches, respectively, and total scores of ≥ 50 indicate the need for medical care and specialist consultation [19]. In this study, patients with KHIT-6 scores of ≥ 50 who therefore required medical care were targeted. A study by Kosinski et al. [22] revealed that the HIT-6 had an internal consistency of 0.89 and Cronbach’s alpha (α) values of 0.80 and 0.90. Headache intensity was evaluated at baseline and 5 and 10 weeks post-intervention.

### Cervical function and pain level

#### Cervical kyphotic angles

A radiographic imaging device (PRIMA; Fujifilm Co., Tokyo, Japan) was used to measure the cervical kyphotic angle. Depending on the patient’s age, sex, and physique, the exposure was administered for 0.02 s at 200 mA, 70–80 kV, and at a distance of approximately 2 m. The images were captured using digital films.

**Fig. 2:**
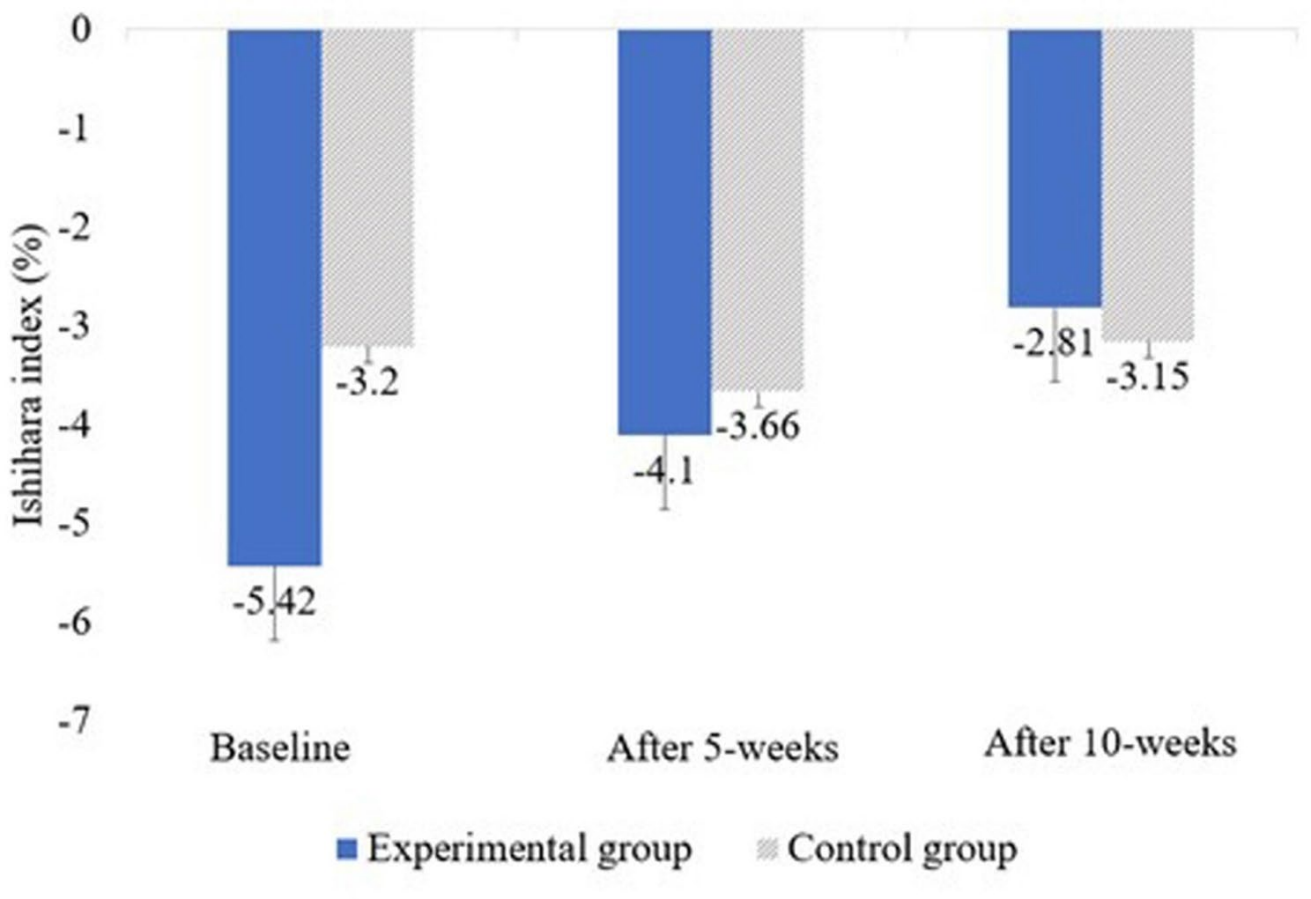
Formula for calculating the Ishihara index using radiographic images: (Ishihara index (%) = ([a1 + a2 + a3 + a4] / A) × 100)

The patients’ radiographs were obtained with the Frankfurt plane parallel to the floor and the jaw in the intercuspal position. The average value of the angles measured from the imaging data was used for the analysis (Fig. 2).

The cervical kyphotic angle was measured at baseline and 5 and 10 weeks post-intervention based on the Ishihara index (ISHIHAR-I). The category of 5–25% is defined as the normal range; >25% is defined as excessive forward bending; 0–5% is defined as straightening; and < 0% is defined as kyphosis [23]. Takeshita et al. [23] demonstrated a significant correlation (95% confidence interval [CI]: 0.94–0.96) between ISHIHAR-I and the cervical kyphotic angle (C2–7).

#### NDI

The NDI is a self-rated evaluation tool used to assess the limitations in daily life due to NP. Individual item scores range from 0 (no disability) to 5 (total disability), with a total possible score of 50. Higher total scores indicate a greater severity of neck disorder. Scores of 0–4, 5–14, 15–24, 25–34, and ≥ 35 are classified as no, mild, moderate, severe, and complete disability, respectively. Vernon and Mior [24] identified eight studies and measured the test-retest reliability of the NDI with a high consistency of 0.90–0.93. Furthermore, results from seven of the studies revealed that the measured Cronbach’s α values ranged from 0.74 to 0.93. The NDI was measured at baseline and 5 and 10 weeks post-intervention..

### NP intensity

NP intensity was assessed based on the intensity of pain experienced by the participants during the study using the numerical rating scale (NRS). The NRS is a pain measurement tool developed by Sartain and Barry [25] that presents a single question, after which the current pain is scored numerically. Its advantage is that its reliability (r = 0.90) is verified, allowing pain evaluation to be conducted in the outpatient department or over the phone. The scores are assigned as follows: 0, no pain; 1–3, mild pain; 4–6, moderate pain; and 7–10, severe pain. Higher scores indicate more severe pain. The NP level was evaluated at baseline and 5 and 10 weeks post-intervention.

### Jaw function and pain intensity: JFLS-8

The Jaw Functional Limitation Scale-20 (JFLS-20) evaluates functional jaw impairment. However, this scale includes sexual questions that do not conform to Korean culture; therefore, its short version, the JFLS-8, was used in this study. The internal consistency of the JFLS-20 is reportedly 0.90, whereas that of the JFLS-8 ranges from 0.84 to 0.86, indicating excellent reliability, sensitivity, and validity [26]. Furthermore, the JFLS-8 was abbreviated for the evaluation of TMDs [26]. Each item of the JFLS-8 was coded as 0–3 (0 = 0; 1 = 1–3; 2 = 4–7; and 3 = 8–10) and scored. When coded, the reliability increased from 0.67 to 0.82, and the intra-class correlation coefficient (ICC) was 0.82 [26]. The JFLS-8 was administered at baseline and 5 and 10 weeks post-intervention.

### TMJ PPT assessment

The pressure pain threshold was measured using an algometer (Baseline; Fabrication Enterprises, Inc., Irvington, NY, USA) to assess TMJ pain levels. The device was pointed toward the skin, overlaying a tender point in the TMJ (masseter muscles). The average score was calculated to obtain a single score.

According to Chesterton et al. [27], when this assessment was applied to healthy individuals at 5 N, the ICC was high (ICC: 0.91, 95% CI: 0.82–0.97). However, the ICC was moderate for patients with TMD (ICC: 0.64). The PPT values were measured at baseline and 5 and 10 weeks post-intervention.

## Experimental procedures

In total, 42 individuals with myofascial TMD accompanied by headaches were recruited and 34 with minimum scores ≥ 50 points on the KHIT-6 were selected. The pain intensity of the trigger points in the masseter muscle of these patients was > 30 mm on the 100-mm VAS. The 34 selected patients were assigned to two groups with 17 participants each: the experimental group (comprising patients who received cervical spine-focused manual therapy and stretching exercises) and the control group (comprising patients who underwent conservative physical therapy only). Patients were assigned using a randomized allocation method (www.randomize.org).

The intervention comprised conservative physical therapy for 60 min in both groups and an additional 40 min of cervical spine-focused manual therapy and stretching exercises in the experimental group. Treatment was administered weekly for 10 weeks (10 treatment sessions in total). Interventions were performed by the same physiotherapist with over 20 years of experience in manual therapy. Patients in both groups were evaluated after 5 and 10 weeks of intervention.

## Conservative physical therapy

Conservative physical therapy was initiated in both groups, and it comprised surface heat treatment, infrared therapy, interference current therapy, and ultrasound treatment, once per week for 10 weeks. The procedure was as follows.

## Manual therapy and stretching exercises of the cervical region

The protocols reported by La Touche et al. [18] and Calixtre et al. [4] were used as references. Accordingly, manual therapy directed at the cervical bone was performed. Direct intervention was applied to the cervical spine for approximately 35 min (25 min for manual therapy and 10 min for muscle stabilization exercises), once per week for 10 weeks. In addition, stretching exercises focused on the cervical region were applied for approximately 5 min once per week for 10 weeks. The procedures are described in detail below:

(1) Modified upper and lower cervical flexion mobilization (C0–C4): Patients were asked to lie supine with the cervical spine in a neutral position. The therapist held the occipital bone with the first finger and medial aspect of one hand, while the other hand was placed on the patient’s forehead. The mobilization force was sufficient to bend the upper and lower cervical spines as the hand provided cephalic traction; caudal pressure was applied using the hand on the forehead. This technique promoted flexion of the upper and lower cervical spines (C0–C4), at a slow rate of 2 s per oscillation (0–5 Hz) for a total of 10 min. In a previous study, this cervical flexion mobilization was used for the upper cervical spine [18]. Furthermore, the authors’ previous study [14]revealed that the shape of the cervical spine is usually kyphotic at the C3–C4 region in patients with TMD. Therefore, an additional procedure was performed on the C3–C4 region.

(2) Modified central posterior-anterior mobilization (C4–C5) [18]: Patients were asked to lie prone with the C1–C4 vertebrae in a neutral position. The therapist placed the tip of the thumb on the posterior surface of the C4–C5 spinous process, one thumb each on the C4–C5 spinous processes, and the other fingers were gently placed around the neck. Mobilization was applied at a slow rate of two oscillations per second (2 Hz). This procedure was performed three times and lasted for 3 min each time, with intervening 1-min rest periods; therefore, the total procedure time was 11 min. In a previous study [18], only the lower cervical region (C5) was treated; however, in this study, C4 was also included in the treatment based on evidence that a kyphotic shape is commonly observed at the C4 level in patients with TMD [14].

(3) Craniocervical flexor stabilization exercises: For the craniocervical flexor stabilization exercises, we followed the protocol described by Falla et al. [28], which focuses on the deep cervical flexor muscles. Craniocervical flexion, involving neck and head flexion, was performed with the patient in the supine position. Furthermore, the head was maintained in contact with the supporting surface to facilitate activation of the deep craniocervical flexor musculature (particularly the longus capitis muscle), with minimal activity of the superficial cervical flexors (sternocleidomastoid and scalene muscles) [29]. The contraction was confirmed using a pressure sensor (Stabilizer; Chattanooga Group, Inc., Chattanooga, TN, USA). In addition, the patients were instructed to maintain the pressure using visual feedback for 10 s, with no contraction of the superficial neck flexor muscles. The therapist assessed the condition of the muscles by facilitation and confirmed that the patient could maintain a pressure of 20–22 mmHg at the target level of the head–neck flexion for 10 s, without flexing the superficial neck muscles or making any sudden movement. The contraction was repeated 10 times every 10 s, with a 10-s interval between each contraction. The number and duration of each set of contractions remained constant. Falla et al. [30] demonstrated that the craniocervical flexion test accompanied increased electromyographic activity in the deep cervical flexor muscles.

(4) Sustained natural apophyseal glide for headache [31]: The patient sat on a chair beside the therapist. The therapist placed the right index, middle, and ring fingers at the base of the occiput, while the middle finger of the same hand and the little finger lay over the C2 spinous process. The therapist’s right little finger was subsequently placed over the lateral border of the thenar eminence, and gentle pressure was applied (via the thenar eminence over the little finger) in a ventral direction on the spinous process of C2, whereas the skull remained under the control of the therapist’s right forearm. The pressure applied by the index finger moved the lower vertebra forward under the first vertebra until the slack was taken up. Consequently, the first vertebra moved forward under the base of the skull. This vertebra was quickly moved forward until the end range was palpable, and this position was maintained for at least 10 s. This procedure was repeated for 5 min. A study by Hall et al. [31] revealed that performing sustained natural apophyseal glides on patients with a headache of upper cervical origin significantly increased the flexion of the neck bone by 15° (p < 0.001).

(5) Stretching exercises in the cervical region: The upper trapezius, scalene, semispinalis capitis, splenius capitis, and sternocleidomastoid muscles are directly involved in head positioning. Misalignment of the head and neck has been reported when these muscles are shortened due to contraction [32]. Stretching exercises for the abovementioned muscles were performed with the patients seated. Each stretch was performed according to patient perception (a score of 8 on a scale of 0–10: 0, no stretching; 10, the maximum height of the muscle) at high intensity for 25–30 s.

### Statistical analysis

SPSS (version 25.0; IBM Corp., Armonk, NY, USA) was used for all statistical analyses. Descriptive statistics (means and standard deviations) and frequency analyses were used to assess the baseline characteristics of patients. The Shapiro–Wilk test was performed to determine data distribution normality in both groups. Repeated-measures analysis of variance, with time (pre-intervention, after 5 and 10 weeks of intervention) as the within-subject variable, was performed to investigate the effect of cervical spine-focused manual therapy and stretching exercises on pain and disability in patients with myofascial TMD accompanied by headaches. The Bonferroni test was conducted for post-hoc analysis; p < 0.05 was considered statistically significant for all analyses.

## Results

### Baseline patient characteristics

Baseline characteristics of the patients are presented in Table 1. The average duration of symptoms in patients with myofascial TMD headache was 4.1 (95% CI: 1.5–10.2) years. All patients were right-handed, and none of them received medications during the study period.

**Table 1:** Patient characteristics

Variables	EG (n = 17)	CG (n_2_ = 17)	t/χ^2^
Sex (male/female)	5/12^a^	2/15	0.000
Age (years)	34.47 ± 10.51^b^	37.59 ± 15.16	0.063
Height (cm)	163.00 ± 9.34	164.06 ± 6.39	0.141
Weight (kg)	58.35 ± 11.47	58.82 ± 9.36	0.234
BMI^c^ (kg/m^2^)	21.81 ± 2.91	21.86 ± 3.31	0.233

EG: Experimental group, CG: Control group, ^a^Numbers, ^b^Mean ± standard deviation, ^c^BMI: Body mass index

## Between-group comparison of changes in pain levels

Findings of the between-group comparison of the changes in pain levels after the interventions are presented in Table 2.

**Table 2:** Changes in headache, neck pain intensity, and TMJ pain levels between the two groups

Variables		EG (n = 17)	CG (n = 17)	t	F (Group × time)
KHIT-6^b^	Baseline	61.53 ± 8.04^a^	59.88 ± 8.03	0.597	12.015^**^
	5 weeks	57.29 ± 6.62^†^	60.06 ± 7.72	-1.120	
	10 weeks	48.88 ± 9.76^†^	59.82 ± 6.88	-3.780^**^	
	F	13.745^**^	0.201		
NRS^c^	Baseline	7.82 ± 1.51	7.53 ± 1.41	0.585	29.219^**^
	5 weeks	6.12 ± 1.21^†^	7.53 ± 1.23	-3.361^**^	
	10 weeks	4.24 ± 1.48^†,‡^	7.24 ± 1.20	-6.490^**^	
	F	49.892^**^	1.995		
PPT^d^	Baseline (L/R)	1.19 ± 0.43/ 1.39 ± 0.46	1.17 ± 0.42/ 1.45 ± 0.48	0.079/ -0.360	21.933^**^/ 15.137^**^
	5 weeks (L/R)	1.35 ± 0.37^†^/ 1.50 ± 0.44^†^	1.18 ± 0.43/ 1.44 ± 0.48	1.177/ 0.424	
	10 weeks (L/R)	1.47 ± 0.37^†,‡^ 1.60 ± 0.45^†^	1.16 ± 0.42/ 1.47 ± 0.48	2.210^*^/ 0.824	
	F (L/R)	25.708^**^/19.666^**^	0.397/3.782^*^		

EG: Experimental group, CG: Control group, ^a^Mean ± standard deviation, ^b^Korean Headache Impact Test-6 (range: 36–78), ^c^Neck pain intensity (range: 0–10), ^d^Temporomandibular joint pain pressure threshold, ^†^There is a significant difference from the baseline (p < 0.05), ^‡^There is a significant difference from 5 weeks after intervention (p < 0.05), ^*^p < 0.05, ^**^p < 0.01

A greater reduction was noted in the intensity of headaches, NP, and TMJ pain evaluated after 10 weeks of intervention in the experimental group than in the control group (p < 0.01). Additionally, the headache, NP severity, and TMJ pain levels in the experimental group decreased significantly from their baseline values (p < 0.01) after 5 and 10 weeks of intervention. NP intensity and left TMJ pain showed significant reduction after 10 weeks of intervention compared with those after 5 weeks of intervention (p < 0.01). In the control group, the right TMJ PPT showed a significant decrease after 10 weeks of intervention (p < 0.05; Table 2).

## Between-group comparison of changes in neck dysfunction

Findings of the between-group comparison of changes in pain and neck dysfunction levels after the intervention period are presented in Table 3.

**Table 3:** Changes in neck dysfunction in the experimental and control groups

Variables		EG (n = 17)	CG (n = 17)	t	F (Group × time)
ISHIHAR-I^b^	Baseline	-5.42 ± 6.14^a^	-3.19 ± 6.94	-0.988	8.205^**^
	5 weeks	-4.09 ± 5.65^†^	-3.66 ± 6.96	-0.200	
	10 weeks	-2.80 ± 4.92^†^	-3.15 ± 6.97	0.166	
	F	10.142^**^	1.314		
NDI^c^	Baseline	30.94 ± 8.05	32.88 ± 7.39	-0.732	22.092^**^
	5 weeks	25.12 ± 7.56^†^	32.59 ± 6.97	-2.29^**^	
	10 weeks	15.94 ± 7.73^†,‡^	31.88 ± 6.61	-6.459^**^	
	F	27.902^**^	4.026^*^		
JFLS-8^d^	Baseline	14.12 ± 4.07	16.35 ± 2.31	-1.966	15.232^**^
	5 weeks	12.18 ± 3.57^†^	16.18 ± 2.24	-3.098^**^	
	10 weeks	10.29 ± 3.21^†,‡^	15.29 ± 1.64^†^	-5.703^**^	
	F	36.054^**^	9.518^**^		

EG: Experimental group, CG: Control group, ^a^Mean ± standard deviation, ^b^Cervical kyphotic angle, ^c^Neck Disability Index (range: 0–50), ^d^Korean Jaw Functional Limitation Scale-8 (range: 0–48), ^†^There is a significant difference from the baseline (p < 0.05), ^‡^There is a significant difference from 5 weeks after intervention (p < 0.05), ^*^p < 0.05, ^**^p < 0.01.

## Discussion

The results of our study demonstrated that patients with myofascial TMD accompanied by headaches who received manual therapy and stretching exercise therapy for the cervical spine experienced significant treatment duration-related reductions in headache severity, NP intensity, and TMJ pain (p < 0.01) after the intervention. Significant between-group interactions were observed in terms of changes in the cervical spine kyphotic angle (ISHIHAR-I), neck disability level (NDI), and JFLS-8 scores (p < 0.01). The level of neck dysfunction improvement was also statistically significant (p < 0.01). Although limited improvements were observed in the control group, conservative physical therapy significantly reduced the PPT, NDI, and JFLS levels after 10 weeks of intervention. This can be understood in the context of a study by Suvinen et al. [33], who reported that 81% of patients with TMD experienced a significant subjective and objective improvement in symptoms and functions through physical therapy.

The DC/TMD includes a new classification called headache attributed to TMD (HATMD), indicating that myalgia and TMJ arthralgia are related to headaches [20]. The International Classification of Headache Disorders, Third Edition Beta (ICHD-3 beta) describes headaches and facial pain caused by problems with TMJ, masticatory muscles, and/or related structures as secondary headaches [34]. Secondary headaches caused by masticatory muscle pain and TMJ joint pain, which are classified and described in these two diagnostic criteria, probably refer to the same condition [35].

The DC/TMD suggests that headaches originate from a myofascial trigger point, induced during palpation of jaw joint muscles and extensive jaw movement, and not from the intracranial structure [13, 35]. According to the results of a systematic review by Armijo-Olivo et al. [36], although the evidence for manual therapy is limited, it has shown significant results in treating myogenous, arthrogenous, and mixed TMDs. Kalamir et al. [37] reported that manual therapy, which targets the oral facial region in myogenous TMD, improves mouth opening and reduces jaw pain compared to botulinum toxin, indicating manual therapy effectively treats myogenous TMD. Ferrillo et al. [38] suggested in a systematic review that manual therapy is effective in treating myogenous TMD, while laser and occlusal splints are mainly effective in relieving pain in arthogeonus TMD. These studies suggest manual therapy is effective mainly in myogenous rather than in arthrogenous TMD; therefore, only patients with myogenous TMD were included in this study.

**Fig. 3:**
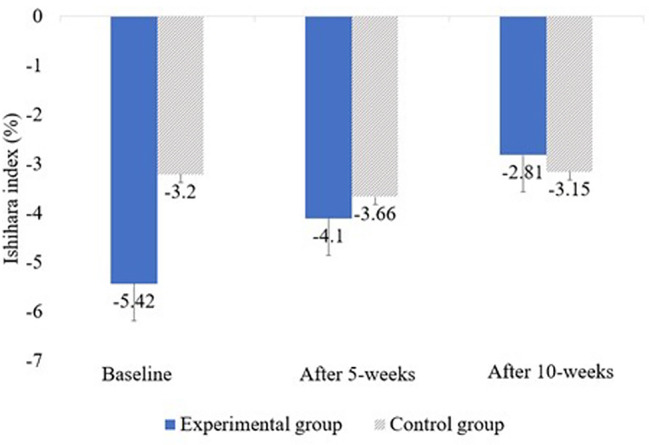
Changes in the cervical kyphotic angle after intervention

In this study, we also looked into changes in the cervical kyphotic angle to improve the diagnosis of the presumed cause of headache secondary to TMJ dysfunction. The causes of “turtle neck”, “straight neck” and “cervical kyphotic angle” include trauma and muscle tension, neck disc, post-neck facet joint syndrome, long-term neck flexion, and secondary phenomena due to thoracic or lumbar deformation [39]. This position causes a forward head posture, so that the center of gravity of the head is located forward [40] on the vertical axis, and increases the load on the posterior neck muscles. This affects the muscles, tendons, and ligaments of the neck region, which can lead to muscular imbalance, and this biomechanical strain can weaken the core stabilization of the neck muscle system and worsen forward head posture-related symptoms [41, 42]. Accordingly, it is known to lead to a cervical kyphotic angle of the neck bone [43]. People with TMDs, often have a cervical kyphotic angle of the neck bone, but it is difficult to presume that a cervical kyphotic angle of the neck bone occurs due to TMDs. This may be a secondary phenomenon due to causes that affect both TMD and cervical kyphotic angle of the neck bone [44, 45]. However, our study aimed to see if manual therapy can improve the cervical kyphotic angle of the neck bone, induce morphological changes in the neck bone, and improve pain and dysfunction. After intervention the authors observed an average increase of 2.62% (measured value by the Ishihara index) in the cervical kyphotic angle (i.e., anterior bending) (Fig. 3). Since this study used a single-center cohort design with a short experiment duration (i.e., 10 weeks), we could not determine whether the results were directly attributable to the treatment of the cervical spine or other variables. However, the findings suggested that manual therapy and stretching exercises can be applied clinically to induce morphological changes in the cervical spine and improve pain and neck dysfunction.

De Laat et al. [46] reported that upper cervical spine movement was limited in patients with TMD. According to the current International Classification of Diseases guidelines, headaches are classified into 300 different subtypes. Among these, physical therapy is very effective for cervicogenic headaches [47]. La Touche et al. [18] applied upper cervical (C1–C2) flexion mobilization based on the association between cervicogenic headaches and the upper cervical spine. O’Leary et al. [48] stated that applying a craniocervical flexor exercise protocol induces an immediate local hypoalgesic inhibition response in patients with NP. Therefore, various researchers consider it appropriate to apply manual therapy to the neck area, particularly the upper cervical spine [4, 17].

However, in a previous study [14], the authors noted that the typical shape of the cervical spine in patients with TMD exhibited similar variations in the C3–C4 region. Hence, the authors modified the manual therapy to the lower part of the cervical spine by additionally applying it to C3 and C4. Distinct morphological changes (an average increase of 2.62% in the cervical kyphotic angle) were noted in the results obtained for the cervical spine levels C3 and C4.

There were no statistically significant results confirming whether the increase in the cervical kyphotic angle correlated with headache, neck pain, and TMJ pain levels; however, the results showed a significant reduction in headache, neck pain, TMJ pain, cervical dysfunction, and JFLS levels after 10 treatments. Therefore, the PPT levels for the masseter muscles and headaches indicate that morphological changes induced by cervical spine treatment can produce hypoalgesic effects.

To the best of our knowledge, no study has demonstrated the activation of descending inhibitory pathways or the occurrence of bilateral hypoalgesic effects in the trigeminal region after applying cervical spine-focused manual therapy and stretching exercises in patients with myofascial TMD accompanied by headaches [18]. However, the authors’ previous study reported a positive correlation between the cervical kyphotic angle and TMJ PPT and a negative correlation between the current and usual pain intensity levels at the TMJ in patients with TMDs [14]. These results suggest that changes in the cervical kyphotic angle can relieve or worsen TMJ symptoms. Moreover, the interaction of the trigeminal nerves and the cervical spine can also increase the incidence of headaches by causing hyperalgesia and allodynia. Therefore, TMD-related headaches, which may have a structural cause, can be influenced by changes in the shape of the cervical spine.

Generally, manual therapy, including TMJ mobilization and the soft tissue technique [49], improves TMJ function and reduces pain when applied to the cervical spine. This procedure alleviates pain via the neurological mechanisms responsible for reducing muscle activity, which may be due to the neuroanatomical connection and biomechanical relationship between these two components of the trigeminocervical complex [50]. In addition, previous studies demonstrated that the application of manual therapy or mobilization of the cervical spine could positively affect pain intensity in patients with TMD [33].

The manual therapy and stretching exercises performed in this study may have improved TMD-related headaches through biomechanical changes in the cervical spine. Alternatively, the patients’ symptoms may have simply improved over time. However, in comparing the duration of the study to the average duration of patient symptoms (4.1 years, 95% CI: 1.5–10.2 years), it is unlikely that the pain would have significantly improved over time without the intervention.

Our study results showed that manual therapy and cervical spine stretching exercises were associated with improvements in the function and pain of patients with TMDs and improvements in the biomechanics of the cervical spine. Pain and disability were successfully alleviated with physical therapy designed for TMD symptoms accompanied by headaches. These study results will improve our understanding of the biomechanical basis of TMDs accompanied by headaches.

Nevertheless, this study has some limitations. First, the control group received 40 min less total therapy per week than the experimental group. In future studies, to better evaluate the significance of between-group differences, the control group should receive the same duration of therapy as the experimental group. Second, the therapist and patients were not blinded because of the patients’ control of the medication. Third, changes in the cervical kyphotic angle of the neck bone, headaches, TMD symptoms, and pain significantly improved after therapy, but future studies are needed to determine whether this is a minimal clinical importance difference that can be recognized and valued by patients.

## Conclusion

The application of manual therapy and cervical spine stretching exercises improved the intensity of headaches, NP, and TMJ pain evaluated after 10 weeks of intervention in the experimental group compared to that in the control group. Additionally, the headache, NP severity, TMJ pain levels, and neck dysfunction in the experimental group decreased significantly from their baseline values after 5 and 10 weeks of intervention. After 10 weeks of intervention, the cervical spine kyphotic angle and neck disability levels decreased significantly more in the experimental group than in the control group. The current findings are significant as they suggest the possibility of pain recovery and improvement of function by achieving structural changes in the cervical spine in patients with TMDs accompanied by headaches. Moreover, these findings may guide protocols and clinical trials involving manual therapy that align morphological structures.

## Data Availability

The datasets used and/or analysed during the current study are available from the corresponding author on reasonable request.

## List of abbreviations

ICC: Intra-class correlation coefficient
JFLS: Jaw Functional Limitation Scale score
NDI: Neck Disability Index
NP: Neck pain
NRS: Numerical rating scale
TMD: Temporomandibular disorder
TMJ: Temporomandibular joint
VAS: Visual analog scale
